# Comparison of protein immobilization methods with covalent bonding on paper for paper-based enzyme-linked immunosorbent assay

**DOI:** 10.1007/s00216-024-05575-4

**Published:** 2024-10-07

**Authors:** Yang Chen, Kaewta Danchana, Takashi Kaneta

**Affiliations:** Department of Chemistry, Okayama University, 3-1-1 Tsushimanaka, Kita-ku, Okayama, 700-8530 Japan

**Keywords:** Paper-based enzyme-linked immunosorbent assay, ELISA, Immobilization, Covalent bonding, Protein

## Abstract

In this study, two methods were examined to optimize the immobilization of antibodies on paper when conducting a paper-based enzyme-linked immunosorbent assay (P-ELISA). Human IgG, as a test-capture protein, was immobilized on paper via the formation of Schiff bases. Aldehyde groups were introduced onto the surface of the paper via two methods: NaIO_4_ and 3-aminopropyltriethoxysilane (APTS) with glutaraldehyde (APTS-glutaraldehyde). In the assay, horseradish peroxidase-conjugated anti-human IgG (HRP-anti-IgG) binds to the immobilized human IgG, and the colorimetric reaction of 3,3′,5,5′-tetramethylbenzyzine (TMB) produces a blue color in the presence of H_2_O_2_ and HRP-anti-IgG as a model analyte. The immobilization of human IgG, the enzymatic reaction conditions, and the reduction of the chemical bond between the paper surface and immobilized human IgG all were optimized in order to improve both the analytical performance and the stability. In addition, the thickness of the paper was examined to stabilize the analytical signal. Consequently, the APTS-glutaraldehyde method was superior to the NaIO_4_ method in terms of sensitivity and reproducibility. Conversely, the reduction of imine to amine with NaBH_4_ proved to exert only minimal influence on sensitivity and stability, although it tended to degrade reproducibility. We also found that thick paper was preferential when using P-ELISA because a rigid paper substrate prevents distortion of the paper surface that is often caused by repeated washing processes.

**Supplementary Information:**

The online version contains supplementary material available at 10.1007/s00216-024-05575-4.

## Introduction

Enzyme-linked immunosorbent assay (ELISA) has become a popular method of detection because it is highly selective, or specific, to target analytes and it lends a more high-throughput nature to the process [1, 2]. The method is based on an immunological reaction where immobilized antibodies bind to the target analyte. This is followed by an enzymatic reaction where the enzyme catalyzes the conversion of a substrate to a product, which thereby enhances the detection sensitivity. Therefore, ELISA is generally specific and sensitive to the target analyte [3].

There are many types of ELISA that generally include homogeneous, competitive, and sandwich assays [4]. In these methods, different enzymes are conjugated with the antibodies of analytes, such as lysozyme, malate dehydrogenase, or glucose-6-phosphate dehydrogenase. A suitable substrate is selected to enhance the sensitivity of the method, and that is dependent on the enzyme that is linked with either the antigen or the antibody. One of the most frequently employed enzyme-substrate combinations is horseradish peroxidase and 3,3′,5,5′-tetramethylbenzidine [5].

Commercially available ELISA kits are based on sandwich assays that utilize a microtiter plate. The sandwich assay involves binding a target analyte to an immobilized antibody on the walls of each well, which is followed by a probe antibody binding the analyte at a different site from that of the immobilized antibody. If the probe antibody is not linked with the enzyme, a secondary antibody linked with the enzyme recognizes it. As a result, the enzymatic reaction with a substrate determines the amount of probe antibody corresponding to the analyte. A microplate reader is used to measure the absorbance, fluorescence, or chemiluminescence, depending on the properties of the enzymatic product.

However, microplate readers are expensive instruments, and commercially available ELISA kits consume expensive microtiter plates and large volumes of substrate solution. Therefore, only well-equipped laboratories are able to use commercially available ELISA kits. Conversely, the use of a paper-based ELISA (P-ELISA) reduces the cost of tools and detection instruments [6]. For example, Verma et al. estimate the price of a P-ELISA at $2.15 [7], which is much cheaper than a commercially available ELISA kit; an ELISA kit for albumin is roughly $1,000 for 98 wells, which amounts to $10 for one sample. Conversely, P-ELISA necessitates manual data processing, which could lead to errors greater than those of microplate readers. However, P-ELISA is useful for determining a rough range for the concentration of a target analyte. Thus, P-ELISA has attracted much attention as a platform that could be an alternative to the use of microtiter plates.

The P-ELISA was first reported by Chen et al. in 2010. Those researchers used photolithography and used a hydrophobic barrier to restrict the detection zone [6]. They immobilized a target protein in the detection zone via nonspecific adsorption and detected it via the use of either fluorescently labeled or enzyme-labeled antibodies that permits the measurement of a target protein. Many studies use the physical adsorption of proteins [4–15] and bacteria [16] on paper substrates. Paper substrates are frequently coated with chitosan to introduce amino groups on the paper surface [17–21]. Amino groups of chitosan are coupled with glutaraldehyde to immobilize capture proteins.

Conversely, immobilization of proteins by covalent bonding is attractive since it is more stable than the physical adsorption against the washing process when using ELISA, and stability also is a factor in the long-term storage of devices. The immobilization of capture proteins on paper requires modification of the functional groups to produce groups of aldehydes, aminos, or carboxyls. The antibody to a target molecule is immobilized covalently using aldehyde groups that are produced via the oxidation of hydroxyl groups to aldehyde groups with sodium periodate [22, 23]. A silane coupling reagent, 3-aminopropyltriethoxysilane (APTS), is also a conventional reagent that is used to introduce amino groups onto paper [24, 25]. Paper substrates are treated with APTS, followed by a reaction with glutaraldehyde to introduce aldehyde groups onto paper. The aldehyde groups react with the amino groups of proteins to immobilize them onto paper.

Obviously, the immobilization efficiency of capture proteins is one of the key issues that determines the performance of P-ELISA because the sensitivity would be directly influenced by the number of proteins that ultimately is retained on the paper. Therefore, in this study, we investigated the immobilization of proteins using APTS and glutaraldehyde and compared the results with a method that uses sodium periodate. We used horseradish peroxidase-labeled anti-human IgG (HRP-anti-IgG) as a model analyte to compare the sensitivity and stability of these immobilization methods. Although the method cannot be directly applied to real samples because HRP-anti-IgG is not a natural substance, this process permits the assessment of binding onto human IgG immobilized on paper. Human IgG antibody was immobilized on a paper surface modified by APTS with glutaraldehyde and sodium periodate, and the immobilized human IgG was bound to HRP-anti-IgG, which was detected via the enzymatic reaction of HRP. The conditions for the enzymatic reaction and the thickness of the paper substrates were also optimized to obtain the best performance.

## Materials and methods

### Materials

Whatman cellulose papers (Grade 1 CHR sheets (thickness, 0.1 mm; pore size, < 11 μm [26]) and Grade 3MM CHR sheets (thickness, 0.34 mm; pore size, < 6 μm [26])) were purchased from Cytiva (MA, USA). Deionized water (DI water, resistivity greater than 18 MΩ cm) was produced using the Elix water purification system (Direct-Q UV3, Merck, Darmstadt, Germany) and was employed to prepare all aqueous solutions. All chemicals and reagents were of analytical grade. Normal human IgG control antibody (Human-IgG) was purchased from R&D Systems, Inc. (MN, USA) and horseradish peroxidase-conjugated monoclonal antibody to human IgG (HRP-anti-IgG) was purchased from the Yamasa Corporation (Tokyo, Japan). The Tokyo Chemical Industry Co., Ltd. (Tokyo, Japan) supplied the 3-aminopropyltriethoxysilane (APTS), which was dissolved in acetone. Hydrogen peroxide (H_2_O_2_) was purchased from the Kanto Chemical Co., Inc. (Tokyo, Japan). Bovine serum albumin (BSA) and 3,3′,5,5′-tetramethylbenzidine (TMB) were obtained from Sigma-Aldrich (MO, USA). Sodium tetrahydroborate (NaBH_4_), sodium periodate (NaIO_4_), potassium periodate (KIO_4_), and a 25% glutaraldehyde solution were obtained from Fujifilm Wako Pure Chemical Corporation (Osaka, Japan). The blocking buffer to prevent nonspecific binding to the paper substrate contained 10 mM phosphate-buffered saline (PBS, pH 7.4, 0.9 (w/v)% NaCl) with 1 (w/v)% BSA. The washing buffer solution consisted of phosphate-buffered saline (pH 7.4) containing 0.05% Tween 20. The TMB solution for the enzymatic reaction contained 0.45 mM TMB and 0.7 M H_2_O_2_, according to a method previously reported by Wang et al. [27].

### Fabrication and modification of paper

**Fig. 1:**
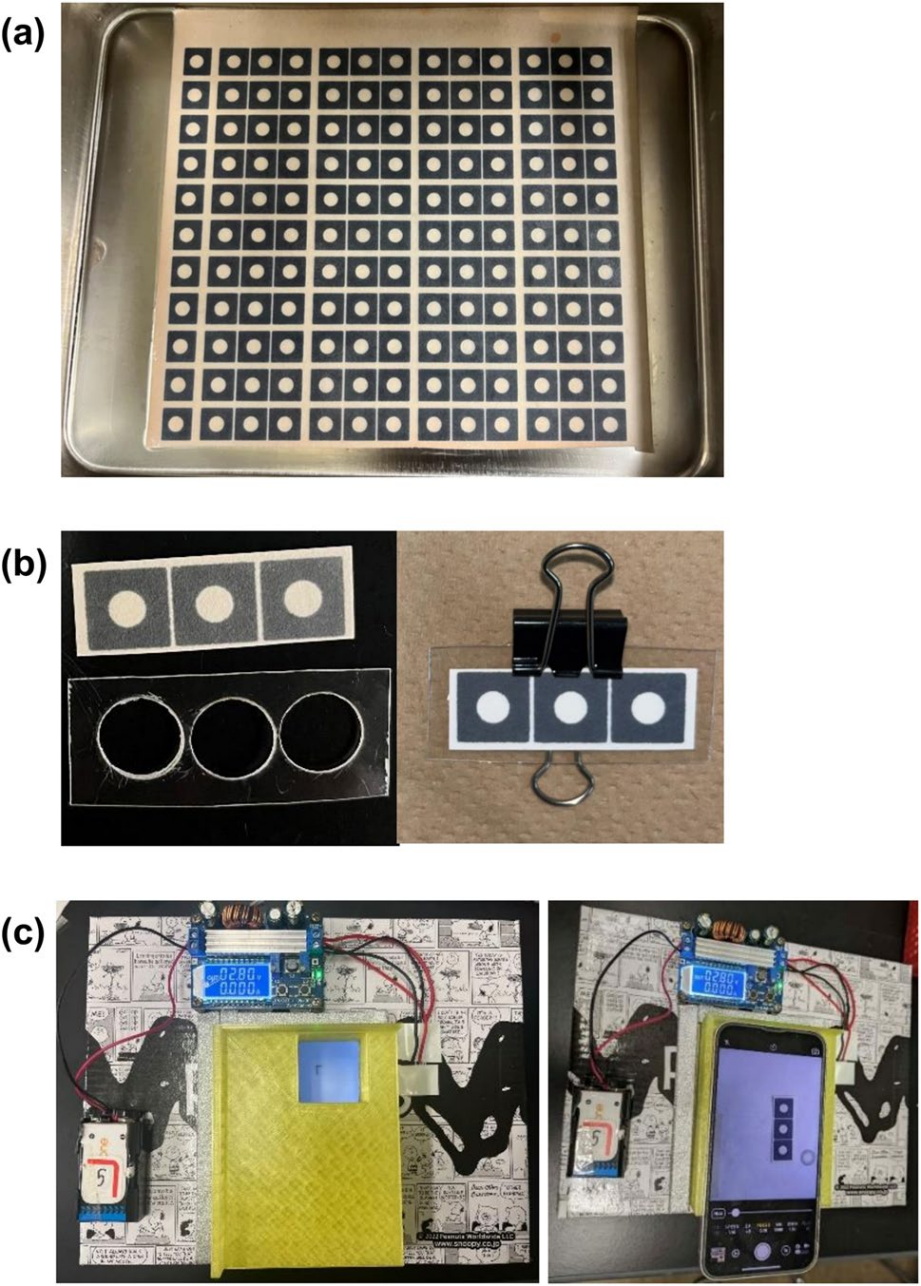
Paper-based analytical devices and an image capture box. **a** Paper sheet treated with APTS and glutaraldehyde. **b** Paper-based analytical device with a holder. **c** Image capture box for a smartphone. The image capture device is equipped with two white light-emitting diodes inside

The P-ELISA devices were designed using PowerPoint 2016 software. The P-ELISA devices were fabricated on filter paper using a wax printer (ColorQube 8580 N, Xerox, CT, USA). The P-ELISA devices were 42 × 15 mm^2^ in size with three circular test zones (diameters of 5 mm), which provided triplicate measurements for each sample, as shown in Fig. 1a. As Fig. 1b shows, the P-ELISA device was fixed on an acrylic plate to prevent bending of the device when loading solutions.

**Fig. 2:**
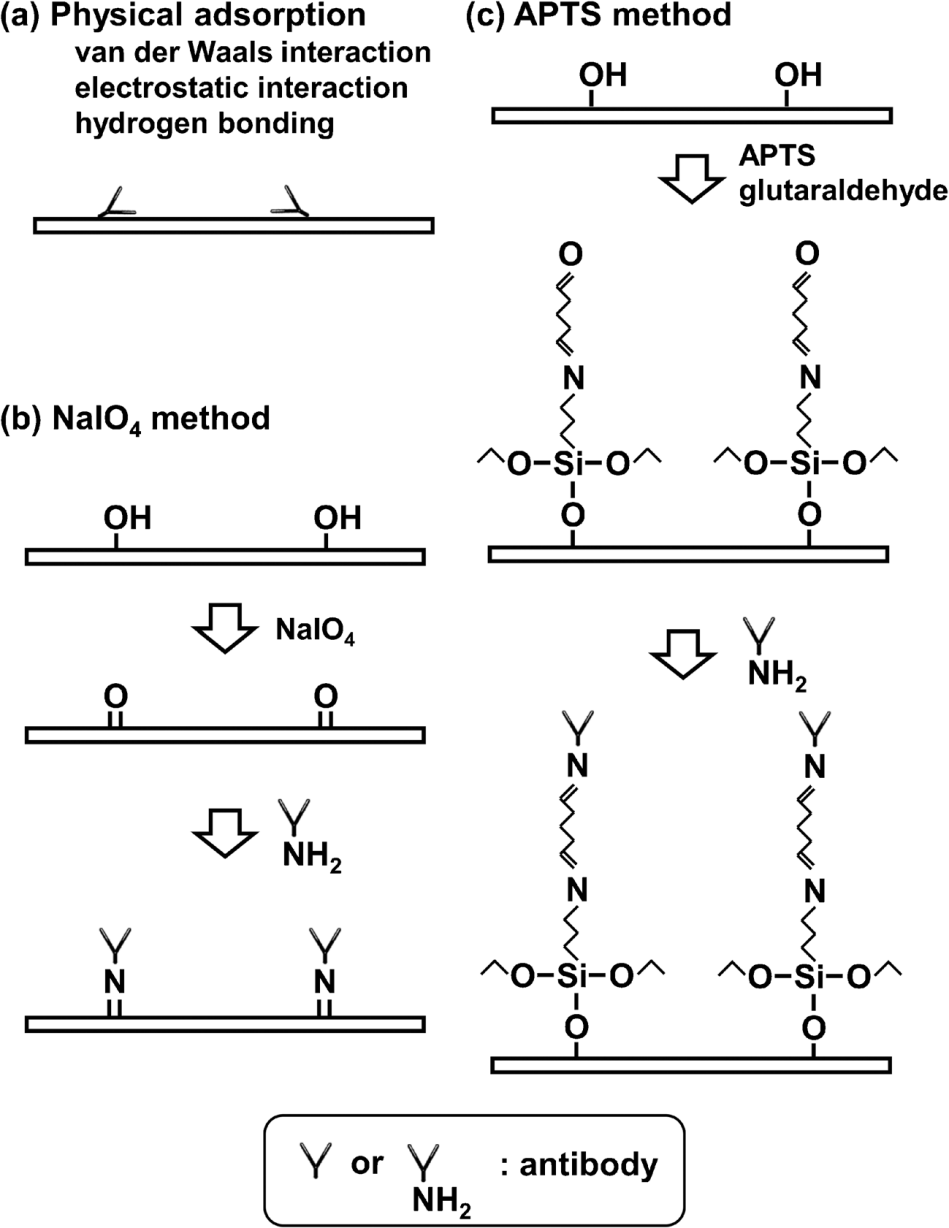
Schematic illustration of immobilization methods

Three immobilization methods are illustrated in Fig. 2; included is a physical adsorption method (Fig. 2a). In this study, two methods were used to immobilize antibodies via covalent bonding: one using NaIO_4_ (Fig. 2b) and another using APTS and glutaraldehyde (Fig. 2c). During modification with NaIO_4_, 10 μL of 0.5 M NaIO_4_ was dropped onto each test zone and allowed to react for 30 min at room temperature in the dark [22]. After the reaction, the unreacted NaIO_4_ was removed by washing with water. In the modification with APTS, filter paper (200 mm × 200 mm, Grade 1 CHR or Grade 3MM CHR) was immersed into a mixture of 6 mL APTS and 60 mL acetone in a square container and allowed to react for 5 h on a lab shaker (80 rpm) at room temperature to produce NH_2_ groups on filter paper. Subsequently, the unreacted APTS was removed via three washes with acetone. Then, the filter paper was dried by heating in an oven (ONW-300S, AS ONE, Tokyo, Japan) at 110 °C for 3 h. The modified paper was printed using a wax printer and heated in an oven at 120 °C for 2 min to allow the wax ink to penetrate the backside of the filter. Grade 1 CHR sheets (thickness, 0.1 mm) were printed on one side whereas Grade 3MM CHR sheets (thickness, 0.34 mm) were printed on both sides with heating for 2 min after printing on one side followed by printing on the other side with heating for 1 min. Finally, the APTS-modified paper was immersed in a 0.05% glutaraldehyde solution for 1 h to generate aldehyde groups on the surface of the filter paper.

### Immobilization of human IgG

Human IgG was immobilized on the modified paper via a Schiff’s base reaction between amino groups of human IgG and aldehyde groups on the surface of the modified paper. Then, a 5-μL aliquot of 0.05 mg mL^−1^ human IgG was dropped into each test zone and allowed to incubate for 20 min at room temperature. After the reaction, the excess human IgG was washed three times with water. A 10-μL aliquot of a blocking buffer containing 1% BSA and phosphate buffer was added to each zone and incubated for 20 min to prevent the nonspecific binding of proteins.

### Testing the stability of immobilized human IgG

During the stability test, the immobilized human IgG was reduced by adding 10 μL of 0.79% freshly prepared NaBH_4_ in 0.1 M of borate buffer (pH 7.3) to convert the imine groups to amine groups, according to a method previously reported by Yoshioka and co-workers [28]. The excess amount of NaBH_4_ was removed via three washes with 10 μL of water. The P-ELISA devices were stored in a refrigerator at 4 °C prior to immunoassay testing.

### Operation of P-ELISA

The analytical procedure for the P-ELISA devices began with the addition of 10 μL of HRP-anti-IgG to three zones of the device, which was followed by incubation for 30 min. Then, 10 μL of the washing buffer solution (phosphate buffer, pH 7.4) was used to wash the zones three times to remove the unbound HRP-anti-IgG. In the washing process, paper pieces placed on the backside of the device accelerated the flow of the washing buffer solution from the frontside to the backside. To measure the amount of bound HRP-anti-IgG, 10 μL of TMB solution was added to each test zone and reacted for 10 min at room temperature to reveal changes in the color of the TMB.

The colorimetric reaction was monitored by images taken with a smartphone. A hand-made box studio was employed to capture the images, as shown in Fig. 1c. The box studio was equipped with two white light-emitting diodes under a cover with a holder to fix the smartphone onto the box. The box studio permitted reproducible and stable illumination to capture images of the P-ELISA devices. The images of the P-ELISA devices were captured before drying them completely because the color of the oxidized TMB disappeared gradually when the incubation time was increased, as previously reported by Busa and co-workers [29].

The captured images were analyzed using ImageJ software, and the average red-green-blue (RGB) values were obtained from the center of each test zone under the analysis type of the RGB stack. An area comprised of 26×26 pixels at the center of the test zones was selected to measure the RGB values. The RGB values were expressed via the ΔRGB, which was calculated by subtracting the measured value from 255 (the maximum value of RGB). The error bars in the graphs correspond to the standard deviation of three measurements.

## Results and discussion

### Optimizing the colorimetric reaction

For the colorimetric enzymatic reactions employed in this study, the color intensity depended on the reaction time and the amount of TMB. We varied the volume of the TMB solution to be added to the test zones after a reaction with 0.5 μg mL^−1^ HRP-anti-IgG, during which the color intensity was monitored every 2 min to optimize the reaction time. The P-ELISA device for this experiment was prepared using the APTS-glutaraldehyde method with Grade 1 paper. Different volumes of TMB solution, 4, 7, and 10 μL, were added to generate color in the oxidized TMB (Supplementary material, Fig. S1). It should be noted that 10 μL is the maximum volume of a solution that could be held in the hydrophilic test zone. The maximum color intensity was generated in 4 min and gradually decreased after 4 min when adding 4 μL of the TMB solution. Conversely, the addition of 7 μL represents the maximum value at 10 min, and the color intensity began to decrease after 10 min. When the TMB volume was 10 μL, the color intensity reached its maximum and remained constant.

The decrease in color intensity is attributed to the disappearance of the color with the evaporation of water. The oxidized TMB, which is the colored product, bleaches when being completely dried. Therefore, in volumes of 4 and 7 μL, water began to evaporate at 4 and 10 min, respectively, which resulted in the initiation of color disappearance. Therefore, 10 μL of TMB maintained a stable intensity even after 10 min because this was the volume that was sufficient to moisturize the test zone.

### Comparison of immobilization methods

The amount of immobilized protein directly influences the sensitivity of P-ELISA. Thus, in the present study, immobilization methods were compared using the reaction between human IgG and HRP-anti-IgG. Physical adsorption is sometimes employed for P-ELISA because it has the advantages of being easy to operate and reducing the cost. However, immobilization via covalent bonding is more stable than physical adsorption, as demonstrated by Zhu and co-workers [22]. Therefore, covalent bonding would be more advantageous in terms of protein immobilization yield, sensitivity, and stability. Thus, two types of modifications were examined in this study, one with NaIO_4_ and the other with APTS and glutaraldehyde, to produce aldehyde groups that are covalently bound with the amino groups of proteins.

**Fig. 3:**
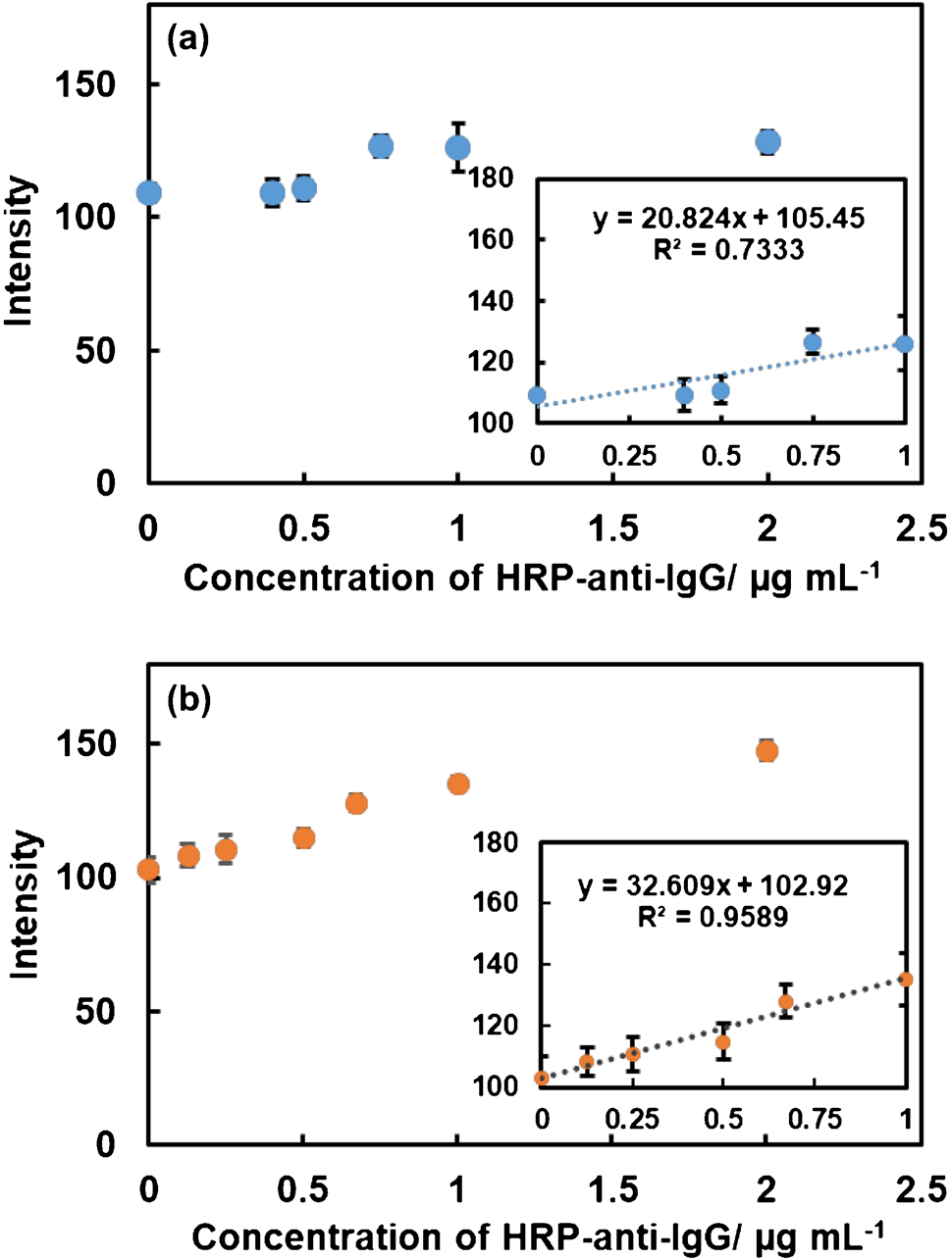
Comparison of two immobilization methods for HRP-anti- IgG assay. **a** Oxidization with NaIO_4_ and **b** APTS and glutaraldehyde. The concentrations of HRP-anti-IgG were 0.4, 0.5, 0.75, 1.0, and 2.0 μg mL^−1^. The error bars indicate the standard deviations for three replicated measurements

In general, a calibration curve of P-ELISA is the relationship between the logarithm of the analyte concentration and the color intensity, and this results in a sigmoidal curve [6, 30]. However, to assess the binding ability of the immobilized human IgG, binding curves were constructed in this study by directly plotting the color intensity against the concentration of HRP-anti-IgG. The dependence of color intensity on the concentration of HRP-anti-IgG is shown in Fig. 3 where the concentration of HRP-anti-IgG was varied within a range of 0.5 to 2.0 μg mL^−1^. Figure 3a and b show the relationship between the concentration of HRP-anti-IgG and the color intensity obtained via immobilization methods using NaIO_4_ and APTS-glutaraldehyde, respectively. In both cases, the color intensity increased with increases in the concentration of HRP-anti-IgG from 0.5 to 1.0 μg mL^−1^. The intensity when using NaIO_4_, however, seemed to be saturated within a range of concentrations of more than 0.5 μg mL^−1^. When comparing the two methods, the APTS-glutaraldehyde method more clearly showed the dependence of the color intensities on the concentration of HRP-anti-IgG. In addition, the correlation coefficient and sensitivity also were larger when using the APTS-glutaraldehyde method compared with the NaIO_4_ method. The slopes, which indicate the sensitivity, can be directly related to the amount of the analyte bound to the immobilized antibodies; therefore, a large slope is preferential to sensitive detection. The limits of detection were estimated to be 0.65 μg mL^−1^ for the NaIO_4_ method and 0.42 μg mL^−1^ for the APTS-glutaraldehyde method. These results imply that the immobilized efficiency of human IgG is greater when using the APTS-glutaraldehyde method. Therefore, the APTS-glutaraldehyde method is preferential to the NaIO_4_ method for P-ELISA in order to obtain the best analytical performance.

### Effect of reduction with NaBH_4_

The Schiff's base reaction generates a relatively unstable imine that must be stabilized by the reduction of imine to amine. In fact, reports in the literature depict how NaBH_4_ reduces imines to amines and stabilizes the immobilized proteins [27, 31]. The effect of reduction was evaluated by comparing the binding curves for the P-ELISA devices with and without treatment with NaBH_4_. The binding curves showed no significant difference in a linear range of 0 to 1 μg mL^−1^, whereas the slope and the correlation coefficient were slightly larger with NaBH_4_ (35.9 and *R*^2^ = 0.9816) than that without NaBH_4_ (32.6 and *R*^2^ = 0.959) (Supplementary material, Fig. S2). Conversely, the standard deviation of each data point was larger with reduction than without, which could be attributed to poor reproducibility in the reduction when using paper. Therefore, these results suggest that the reduction treatment somewhat degrades the reproducibility of P-ELISA because the sensitivity and linear range show a less significant difference.

**Fig. 4:**
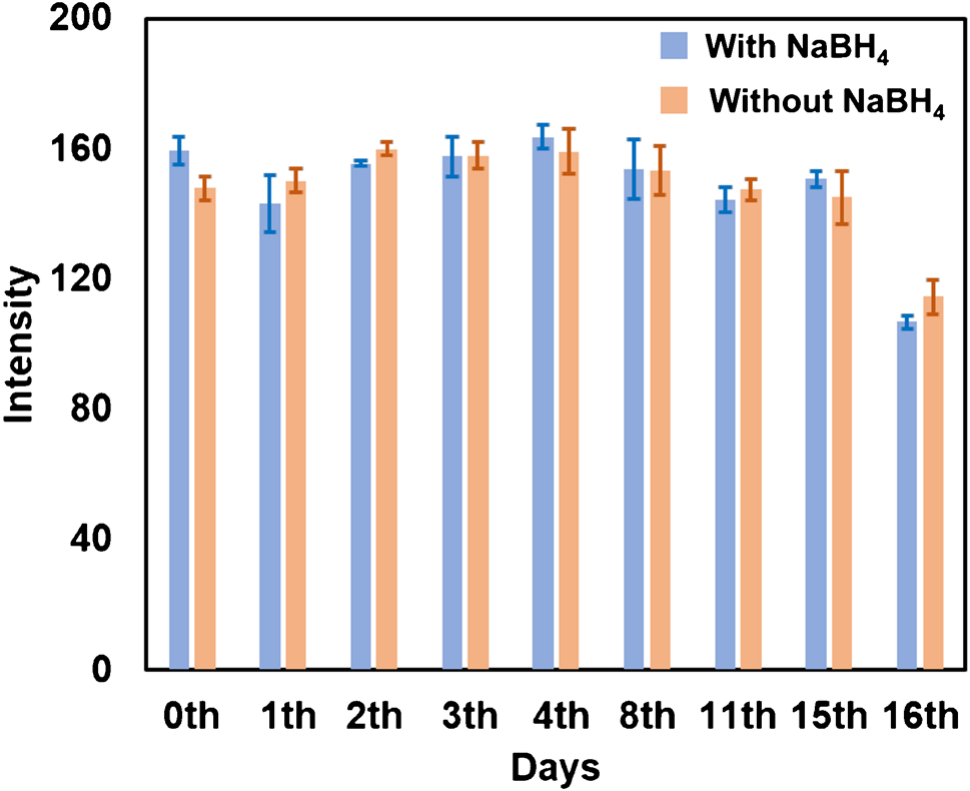
Effect of reduction with NaBH_4_ on the stability of ELISA. The concentration of HRP-anti-IgG, 1.0 μg mL^−1^

The effect of reduction on the stability of the P-ELISA device was also evaluated via immunoassays using 1 μg mL^−1^ HRP-anti-IgG. The P-ELISA devices were prepared on the same day and stored at 4 °C in a refrigerator. The results are shown in Fig. 4. It was apparent that the color intensity seemed to remain unchanged within 15 days for both devices, with and without reduction, and began to decrease after 16 days. We have also confirmed that the color intensity remained stable for only 3 days when the devices were stored at room temperature.

We also used a *t*-test to assess the stability of the device and compared the results of each day with those of the first day. The *t*-test results were slightly different from those obtained via visual assessment, as shown in Fig. 4. The NaBH_4_ treatment showed no significant difference from the first day to the eighth day, but the device appeared to gradually deteriorate from the 11th day. On the other hand, without the NaBH_4_ treatment, there was a significant difference from the first day to the second and third days, but no significant difference from the fourth to the fifteenth day. Since the results from the fourth to the fifteenth day did not show a significant difference in the untreated device, it is likely that the reduction with NaBH_4_ would have less of an impact on stability. Conversely, the significant difference observed in the second and third days should be attributed to the small sample size of the *t*-test (it should normally be larger than 30), the colorimetric reagents, or the experimental operation. Therefore, the reduction of the imine bond to an amine bond between the paper surface and human IgG had no significant influence on the stability of the P-ELISA device.

### Effect of paper thickness

**Fig. 5:**
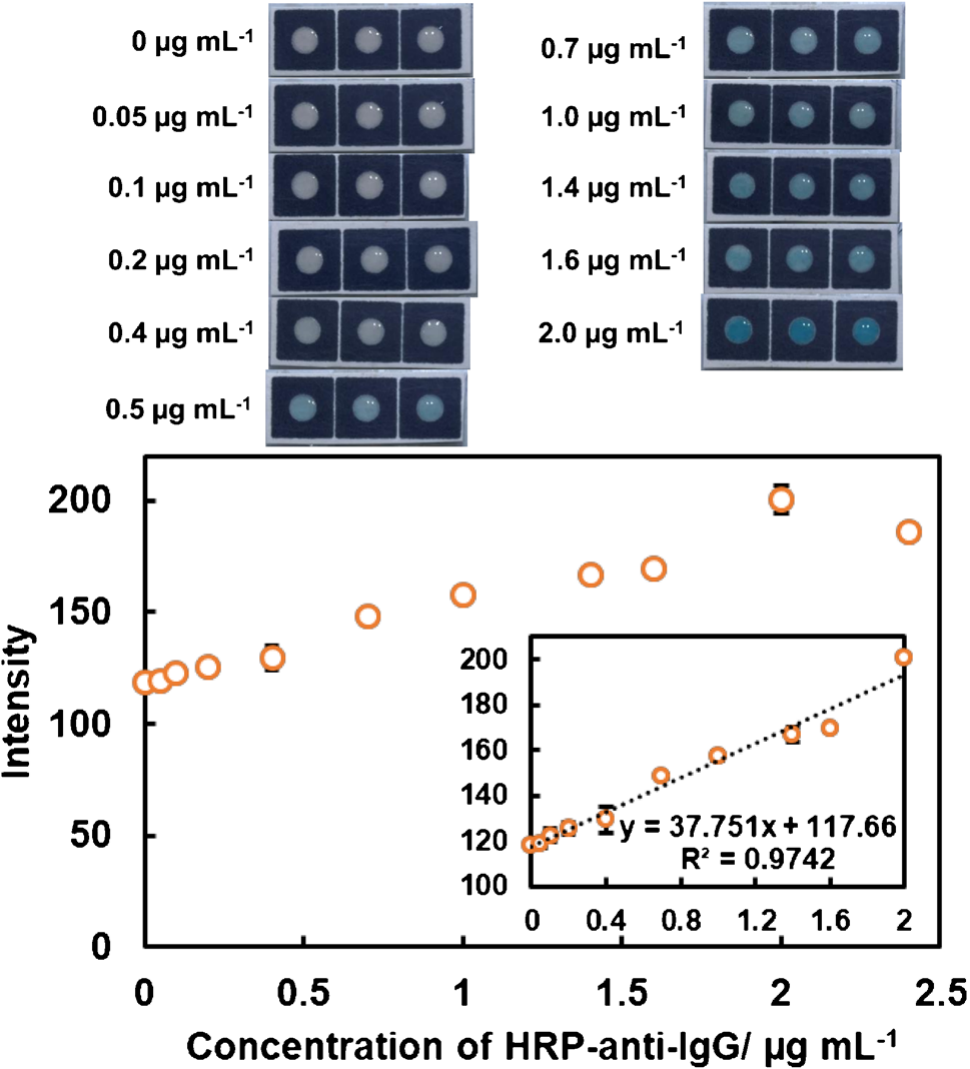
Images of the P-ELISA devices and binding curve for HRP-anti-IgG. Linear range, 0.05–2.0 μg mL^−1^

Two types of paper substrates were compared using the APTS-glutaraldehyde method. Although Grade 1 CHR and Grade 3MM CHR paper represent the same sensitivity (Fig. 3b and Fig. 5), the error bars of the 3MM CHR paper were significantly smaller than those of the Grade 1 CHR. The LOD was also improved to 0.11 μg mL^−1^ when using Grade 3MM CHR paper. The rigidity of the paper substrate contributes to the good reproducibility of Grade 3MM CHR paper. The thin paper substrate, Grade 1 CHR, distorted the surface when it was repeatedly washed with solutions, which resulted in poor reproducibility of the color intensity. However, the thick paper substrate, Grade 3MM CHR, maintained a flat surface after being washed repeatedly. In addition, the linear range was expanded to 2 μg mL^−1^ when using the thick paper. Therefore, the thicker paper is preferential when using P-ELISA devices that must be washed many times.

## Conclusions

In this work, we compared two immobilization methods using NaIO_4_ and APTS-glutaraldehyde to produce aldehyde groups on paper substrates. We established that the APTS-glutaraldehyde method is superior to the NaIO_4_ method in terms of reproducibility and sensitivity. We also found that a reduction with NaBH_4_ showed no difference in stability and instead degraded reproducibility, although it exhibited a slight improvement in sensitivity. The devices could be stored for 15 days at 4 °C in a refrigerator without the need to reduce the imino bond to an amino bond. Good linearity was obtained within a range of 0 to 1.0 μg mL^−1^ of HRP-anti-IgG. The effect of paper thickness was also examined, and a thicker paper substrate proved to be preferential when using P-ELISA due to the rigidity that prevents bending caused by the washing process. The present work should provide useful information to help obtain the best performance when immobilizing antibodies on paper for P-ELISA. The developed device cannot directly be applied to real samples because the analyte used in this study is not found in real samples. However, this immobilization method is expandable to sandwich assays when performing P-ELISA for an analyte in real samples.

## Supplementary Information

Below is the link to the electronic supplementary material.

Supplementary file1 (DOCX 69 KB)
