# Supplementary material for: Comparison of protein immobilization methods with covalent bonding on paper for paper-based enzyme-linked immunosorbent assay
Source: Anal Bioanal Chem. 2024 Oct 7;416(28):6679–86. doi: 10.1007/s00216-024-05575-4 (PMC11541255; doi:10.1007/s00216-024-05575-4)
Supplement: Supplementary file 1 — Supplementary file1 (DOCX 69 KB) [file 216_2024_5575_MOESM1_ESM.docx]

**Supplementary Information**

**Comparison of protein immobilization methods with covalent bonding on paper for paper-based enzyme-linked immunosorbent assay**

**Yang Chen, Kaewta Danchana, and Takashi Kaneta***

**Department of Chemistry, Okayama University, 3-1-1 Tsushimanaka, Kita-ku, Okayama 700-8530, Japan**

*Corresponding authors: kaneta@okayama-u.ac.jp


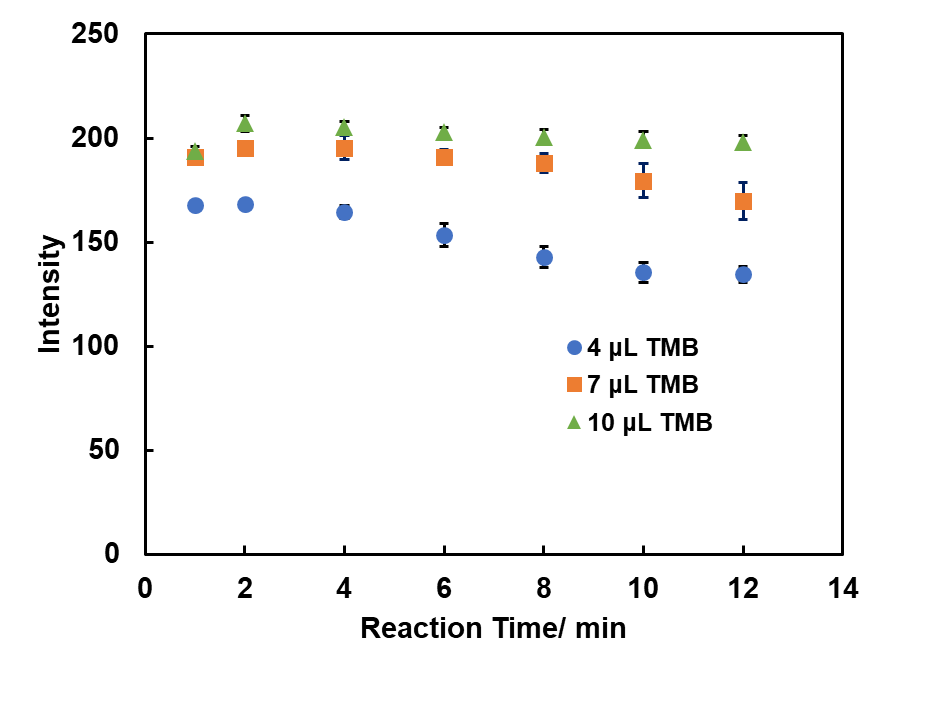


**Fig, S1**: Optimization of the volume of TMB and the reaction time. Volumes of TMB: 4, 7, and 10 μL. Concentration of HRP-anti-IgG, 2 μg mL^-1^. The color signal was captured at a time interval of 2 min.


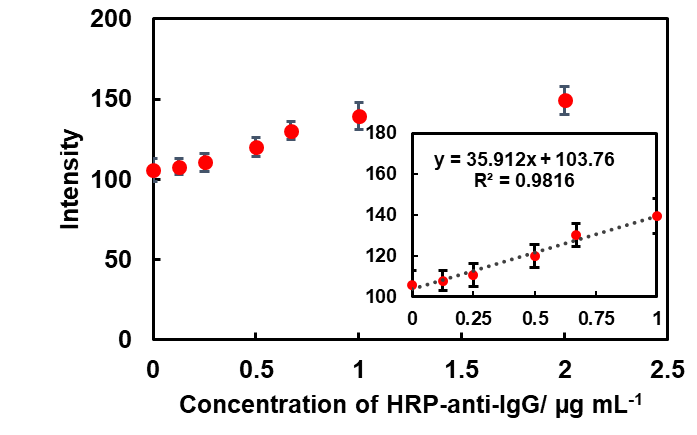


**(a)**

**(b)**

**Fig, S2**: Effect of reduction with NaBH_4_: (a) With reduction; and, (b) without reduction. The concentrations of HRP-anti-IgG: 0.125, 0.25, 0.5, 0.67, 1.0, and 2.0 μg mL-1. The error bars indicate standard deviations for three replicated measurements.
